# Facility-level intervention to improve attendance and adherence among patients on anti-retroviral treatment in Kenya—a quasi-experimental study using time series analysis

**DOI:** 10.1186/1472-6963-13-242

**Published:** 2013-07-01

**Authors:** Patrick Boruett, Dorine Kagai, Susan Njogo, Peter Nguhiu, Christine Awuor, Lillian Gitau, John Chalker, Dennis Ross-Degnan, Rolf Wahlström, Göran Tomson

**Affiliations:** 1 Management Sciences for Health, Nairobi, Kenya; 2 National AIDS & STI Control Programme (NASCOP), Nairobi, Kenya; 3 Management Sciences for Health, Arlington, VA, USA; 4 Harvard Medical School and Harvard Pilgrim Health Care Institute, Boston, MA, USA; 5 Division of Global Health (IHCAR) and Medical Management Centre (MMC), Karolinska Institutet, Stockholm, Sweden

**Keywords:** Appointment-keeping, Medication gaps, Self-reported adherence, Indicators, Monitoring performance

## Abstract

**Background:**

Achieving high rates of adherence to antiretroviral therapy (ART) in resource-poor settings comprises serious, but different, challenges in both the first months of treatment and during the life-long maintenance phase. We measured the impact of a health system-oriented, facility-based intervention to improve clinic attendance and patient adherence.

**Methods:**

This was a quasi-experimental, longitudinal, controlled intervention study using interrupted time series analysis. The intervention consisted of (1) using a clinic appointment diary to track patient attendance and monitor monthly performance; (2) changing the mode of asking for self-reported adherence; (3) training staff on adherence concepts, intervention methods, and use of monitoring data; (4) conducting visits to support facility teams with the implementation.

We conducted the study in 12 rural district hospitals (6 intervention, 6 control) in Kenya and randomly selected 1894 adult patients over 18 years of age in two cohorts: experienced patients on treatment for at least one year, and newly treated patients initiating ART during the study. Outcome measures were: attending the clinic on or before the date of a scheduled appointment, attending within 3 days of a scheduled appointment, reporting perfect adherence, and experiencing a gap in medication supply of more than 14 days.

**Results:**

Among experienced patients, the percentage attending the clinic on or before a scheduled appointment increased in both level (average total increase immediately after intervention) (+5.7%; 95% CI = 2.1, 9.3) and trend (increase per month) (+1.0% per month; 95% CI = 0.6, 1.5) following the intervention, as did the level and trend of those keeping appointments within three days (+4.2%; 95% CI = 1.6, 6.7; and +0.8% per month; 95% CI = 0.6, 1.1, respectively). The relative difference between the intervention and control groups based on the monthly difference in visit rates increased significantly in both level (+6.5; 95% CI = 1.4, 11.6) and trend (1.0% per month; 95% CI = 0.2, 1.8) following the intervention for experienced patients attending the clinic within 3 days of their scheduled appointments.

The decrease in the percentage of experienced patients with a medication gap greater than 14 days approached statistical significance (-11.3%; 95% CI = -22.7, 0.1), and the change seemed to persist over 11 months after the intervention. All facility staff used appointment-keeping data to calculate adherence and discussed outcomes regularly.

**Conclusion:**

The appointment-tracking system and monthly performance monitoring was strengthened, and patient attendance was improved. Scale-up to national level may be considered.

## Background

HIV and AIDS continue to affect millions of people globally, particularly those in sub-Saharan Africa. By December 2010, approximately 432,000 patients in Kenya were receiving antiretroviral therapy (ART), which was an absolute increase of 28% over 2009 figures and represented 61% of those in need [1]. More than 80% of patients in Kenya remain on treatment one year after initiating ART [1,2].

Adherence rates can vary enormously between facilities [3], and poor adherence to long-term therapies compromises treatment effectiveness, making this a critical public health issue [4]. Non-adherence to prescribed ART is one of the strongest predictors of progression to AIDS and death among people living with HIV [5]. It is also associated with the development of drug-resistant viral strains [6].

Among patients on ART, different challenges are faced, both at the patient level and at the system level, during the early/first months of treatment as compared to the maintenance phase. Such challenges may include disclosure, food insecurity, access to HIV Information Education and Counselling material, and stigma. It is known that most adherence failures occur early in the course of treatment [7,8].

Appointment-keeping rates correlate with treatment outcomes (i.e., weight gain and CD4 counts) [9]. Bisson and colleagues showed that using pharmacy refills to assess ART adherence was as accurate as CD4 counts for detecting virological failure [10], while Paterson et al. demonstrated that resistance to antiretrovirals decreased with better adherence [11]. Visiting clinics early after initiation of ART is an independent predictor of long-term clinical progression in HIV-positive patients [7].

Although research has shown that adequate ART adherence rates can be achieved in resource-poor settings [12], there are concerns that adherence to ART in such settings may decline as treatment access increases [13]. Previous multi-country studies in Ethiopia, Kenya, Rwanda, and Uganda have identified the need to strengthen health systems, so that facilities can use routine data to improve adherence and clinic attendance [3,14].

Qualitative studies in Uganda and Ethiopia identified several factors that affect ART adherence. Survey respondents cited lack of appointments, overcrowding, and no effective mechanisms for community outreach to defaulters as key systems failures associated with low adherence [8]. In Kenya, challenges in monitoring adherence include difficulty in tracking patients and identifying defaulters, weak community linkages, shortage of skilled community health workers, lack of adherence monitoring tools, large numbers of patients, and lack of national guidelines on adherence monitoring [15].

The objective of this intervention study was to strengthen clinical appointment and adherence monitoring systems in Kenyan health facilities and measure any effects on the patients’ attendance and adherence to ART using standard indicators [16].

## Methods

### Study design and setting

We conducted a longitudinal cohort study in a convenience sample of 12 rural health facilities situated in Central (2 facilities), Rift Valley (4 facilities), and Eastern (6 facilities) provinces in Kenya. The facilities were sampled from the monitoring and evaluation database maintained by the Kenya National AIDS & STI Control Programme (NASCOP), and was implemented over a one-year period. Healthcare providers at the facilities included medical officers, clinical officers, nurse practitioners, pharmacy practitioners, and records officers.

### Facility sample

The inclusion criteria for sampled health facilities were providing ART for at least one year prior to the study, having between 100 and 500 adult patients on ART, and being located within 200 km from Nairobi. We selected six intervention facilities that met the criteria and an additional six control facilities that were matched to each intervention facility based on geographical location, number of patients on ART, and level of care. Facilities that offered food or other economic support to patients, had ongoing research activities, or that had been affected by the post-election political instability in 2007 were excluded from the study.

### Study population

The study population included patients aged 18 years or more, who were on ART. We also collected data on the intervention implementation process from selected staff at each clinic. The patients selected for the study were allocated to one of two cohorts. The reason for this division of patients was to account for the different challenges faced by staff during the first months of treatment, compared to during the life-long maintenance phase. The first cohort included ART-experienced patients, who had initiated treatment at least one year prior to the study and who had visited the clinic at least once between 13 and 15 months prior to the baseline survey. The second cohort included all newly treated patients recruited month-by-month from 12 months pre-intervention and through the post- intervention period. We analyzed only their first 120 days of treatment.

In each facility, we aimed to sample 100 experienced patients and as many newly treated patients as possible. Patients had all initiated treatment at the facility in question; those who had transferred in or who had prior exposure to ART, such as through prevention of mother-to-child transmission programs, were excluded.

Patients initiated on antiretroviral therapy were scheduled for an appointment in two weeks for first review. Thereafter, time between appointments ranged from one month up to a maximum of three months for clinically stable patients. Medicines were dispensed based on the days until the next clinic visit, with the normal amount of pills lasting between 30 to 90 days.

### Data collection

Trained data collectors with backgrounds in clinical medicine, pharmacy, nursing, or social science abstracted quantitative data from patients’ clinical and pharmacy records. Data were collected in two waves—a baseline survey that took place one month before the start of the intervention, and a follow-up survey 13 months after the start of the intervention. We developed and pretested data collection instruments in two ART facilities prior to beginning the study. All study tools are available on request from the first author.

Data for the cohort of experienced and new patients were extracted from the ART clinic register and individual patient files. We initially sampled 120 records from the ART clinic register, and then selected 100 patients who qualified for the study. We also used the ART register to identify the newly treated cohort of patients who started ART during the period from 12 months prior to the baseline survey date up to 3 months before the date of final data collection. Data on perfect adherence was abstracted from the patient clinical record (MoH-257) based on the patient’s self-reported, recalled perfect adherence during the three days prior to clinic visit.

### Study variables

Variables extracted from the clinic and pharmacy records are included in Table 1. The outcomes for the study included several calculated measures of clinic attendance and treatment adherence: 1) attending the clinic on or before the scheduled date, 2) attending the clinic within 3 days of the scheduled appointment date, 3) reporting perfect adherence, and 4) experiencing a gap in medication supply from the clinic of more than 14 days between recorded visits. In order to obtain the gap in medication supply, the records of dispensing done during each clinic or ART pharmacy visit were reviewed, and the total daily doses of dispensed medicines were recorded for each patient visit.

**Table 1:** Summary of patients’ demographic and clinical characteristics

**Characteristics of study population**	**Experienced cohort **^**1**^			**Newly treated cohort **^**2**^		
**(n = 1894)**	**Intervention**	**Control**	**p-value**	**Intervention**	**Control**	**p-value**
	**(n = 446)**	**(n = 352)**		**(n = 520)**	**(n = 576)**	
Female (n = 1258)	289 (64.8%)	248 (70.5%)	0.0908	337 (64.8%)	384 (66.7%)	0.5171
Mean age (95% CI)	39.4 (38.3–40.5)	38 (37.1–38.9)	0.065	38.6 (37.7–39.5)	39.2 (38.4–40.1)	0.349
*Marital status (%)*						
Single	72 (16.1%)	43 (12.2%)	0.1157	79 (15.2%)	79 (13.7%)	0.1987
Divorced/Widowed	119 (26.7%)	114 (32.4%)		140 (26.9%)	186 (32.3%)	
Married	181 (40.6%)	159 (45.2%)		253 (48.7%)	268 (46.5%)	
*Weight at start of follow-up—mean (95% CI)*						
Male	58.5 (56.7,60.3)	58.3 (56.8,59.9)	0.8974	**54.9 (53.5,56.3)**	**57.3 (56,58.5)**	**0.0144**
Female	54.8 (53.5,56.0)	55 (53.9,56.1)	0.796	52.3 (51.1,53.5)	52.8 (51.7,53.8)	0.5529
*WHO stage at ART initiation (%)*						
Stage 1	**14 (3.1%)**	**46 (13.1%)**	**<0.0001**	**50 (9.6%)**	**88 (15.3%)**	**0.0003**
Stage 2	**61 (13.7%)**	**84 (23.9%)**		**146 (28.1%)**	**199 (34.5%)**	
Stage 3	**278 (62.3%)**	**172 (48.9%)**		**247 (47.5%)**	**224 (38.9%)**	
Stage 4	**31 (7%)**	**22 (6.3%)**		**31 (6%)**	**21 (3.6%)**	
*CD4 value at ART initiation—mean (95% CI)*						
CD4 value	**293 (269.1,316.9)**	**334.3 (309.8,358.8)**	**0.0183**	**169.2 (154.5,183.9)**	**189.4 (176.1,202.7)**	**0.0466**
*Evidence of adherence counselling prior to ART initiation*						
Adherence counselling	**387 (86.8%)**	**238 (67.6%)**	**<0.0001**	**457 (87.9%)**	**401 (69.6%)**	**< 0.0001**

*Missing data: marital status n = 201; WHO staging n = 180; initial CD4 n = 470; initial adherence counseling n = 3.

NB: Figures in bold show significant differences.

### Description of the intervention

The intervention consisted of the following four components:

1) Implementing a clinic appointment diary system for tracking patient attendance. The diary comprised a 12-month record of visits for each patient including the scheduled appointment date and the exact attendance date for each visit. The diary also included an option for the facility to monitor monthly appointment-keeping performance for all of their patients on ART. The diary helped the facilities quickly identify patients who missed appointments and then initiate timely follow-up. Patients who missed appointments were targeted for more focused counselling.

2) Modifying the national routine patient monitoring form (MoH-257) (Additional file 1). The form’s self-reported adherence question, which is asked by trained clinicians during each patient encounter, was changed to read as follows: “Have you missed any medicine in the last three days?” The answer was used to identify patients with less than perfect adherence. Patients reporting missed doses received extra adherence counselling.

3) Conducting targeted training of health service providers drawn from each intervention site. The training curriculum included content on basic adherence concepts adapted from the national adherence training curriculum, introduction to adherence intervention tools, how to extract data to measure attendance-based indicators, and how to analyze and use the data to inform decision-making at the facility.

4) Visiting the facility teams to support their implementation of the intervention. We paid particular attention to helping the facility calculate their monthly appointment-keeping rate (the percentage of patients arriving on or within three days of their appointment) and use the information during their monthly staff meetings.

### Implementation of the intervention

We phased in the intervention, beginning with the first three facilities in March 2008 and following with the next three facilities three months later. This timing was intended to control for any simultaneous external interventions that could influence the study outcomes. The control facilities were not exposed to the intervention, and contact with them was made only at the final evaluation.

The intervention in each facility was implemented over two months. To enhance buy-in among staff, the initial training targeted key people, including the facility in-charge, officer in-charge of the ART clinic, records officer, pharmacist, clinician, and nurse. The training introduced the study tools and disseminated preliminary feedback from the baseline survey. During the training, each facility developed its own intervention implementation plan adapted to their setting. We thereafter made reinforcement visits two weeks after the initial training, and two months after the first reinforcement visit.

Supervision visit were also scheduled three months after the second reinforcement visit for preliminary evaluation of the implementation process. During these visits, the study team reviewed the documentation in the diary and the MoH-257 form, the calculation of the appointment-keeping indicators from the clinic attendance diary, and the recording of self-reported adherence. The study team worked with the comprehensive care clinic team to identify gaps and challenges in the implementation and documented the implementation process, including use of the diary, calculation of indicators, and how indicators were used for monitoring and improvement. The providers did not receive any incentives other than the training and follow-up that was part of the intervention package.

### Data management and analysis

Data extracted from clinical records were entered twice into standardized Excel worksheets, compared and corrected, and then analyzed using Stata v. 11 (StataCorp LP, College Station, Texas, USA). We first compared the characteristics of the intervention and control study groups using frequency distributions and summary statistics. All study outcomes were then expressed as monthly percentages. We used segmented regression analysis to evaluate the effects of the intervention. The regression models included terms to estimate the baseline trend (slope), as well as changes in the level immediately after intervention, and the post intervention trend (slope) of each outcome of interest. A change in level would indicate an immediate effect of the intervention on the outcome indicator of interest, while a change in trend (or slope) of the regression line would point to a longer, more sustained response for the indicator measured. We excluded a two-month lag period following the beginning of the intervention from our models to reflect the time needed for the intervention to take effect (i.e., the expected time until most patients scheduled prior to the intervention would have made a follow-up visit).

Missing data in Table 1 (Demographic characteristics) was highest for CD4 data and WHO staging. Those experienced patients with either missing CD4 or WHO staging data in each study arm did not have significantly different outcome data (change in level or change in trend, of the outcome “Appointment keeping to within 3 days”) compared to the rest of the experienced patients.

Recording of self reported perfect adherence was occasionally missing in the MoH 257 form. Notably, one control facility did not record this outcome consistently, and therefore about 79% of data was missing. In evaluating this specific outcome, only the experienced patients with at least one record of self reported perfect adherence available were considered (328 of 353 in the intervention facilities, and 418 of 446 in the control facilities).

During data collection, all possible attempts were made to ensure that patients’ medicine supply data were collected, but after cleanup and validation, data were missing in 16.2% of patient visit records in control facilities and 12.4% in intervention facilities. It was however noted that pharmacy refills were being issued, in almost all cases, in whole packs of medicines i.e. in multiples of discrete and known numbers based on the quantities of pills in the respective packs, related to the number of weeks remaining until the client’s next clinical appointment. This is commonly done so that the clients have enough medicines to meet their needs until the next clinic visit date. We imputed missing dispensing data based on this assumption.

### Ethical considerations

The Kenyatta National Hospital Ethics and Research Committee granted ethical approval to conduct the study. Patient names were not recorded on any of the data collection forms, and each patient record was assigned a unique identifying number. Written informed consent was sought for all interviews conducted and children were excluded in this study.

## Results

### Demographic characteristics

The study included 1894 patients; the experienced cohort comprised 798 patients (67% female) with a mean age of 39.4 years for intervention sites and 38.0 years for control sites. A significantly greater percentage of experienced patients at the intervention sites (69%) were started on treatment at more advanced stages of illness (World Health Organization [WHO] stages 3–4) compared to control sites (55%). Rates of adherence counselling prior to initiation of ART were significantly higher in the intervention sites (87%) compared to control sites (68%) (Table 1).

The newly treated cohort comprised 1096 patients (66% female), with a mean age of 38.6 years for intervention sites and 39.2 years for control sites. As with experienced patients, a significantly greater percentage of patients in intervention sites (54%) were started on treatment at WHO stage 3–4 compared to the control sites (43%). Adherence counselling prior to initiation of ART in this cohort was also significantly higher in the intervention sites (88%) compared to control sites (70%) (Table 1).

### Appointment keeping

**Figure 1:**
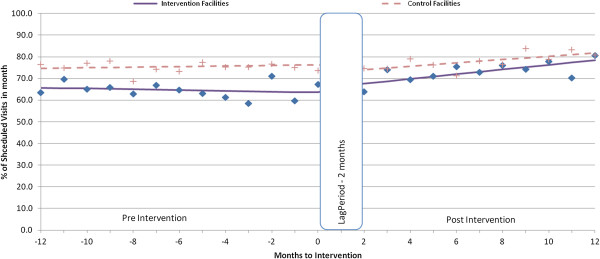
Percentage visits kept or attended before appointment for experienced patients.

**Figure 2:**
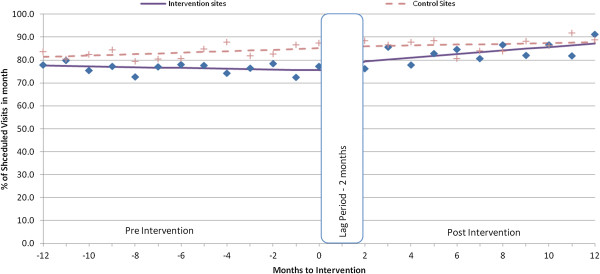
Average % of visits kept within 3 days for experienced patients.

**Figure 3:**
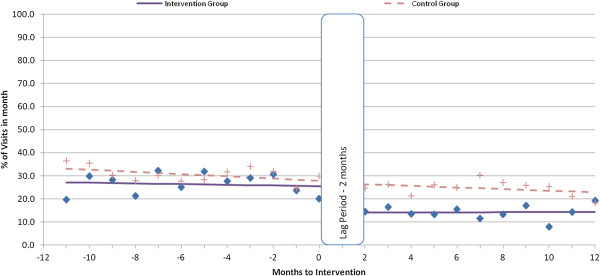
Percentage visits with medication gap of more than 14 days for experienced patients.

Appointment-keeping trends for new and experienced patients at both intervention and control facilities were relatively steady during the pre-intervention period (Figures 1, 2, 3).

Experienced patients had significant increases in both the level (average total increase immediately after intervention) (+5.7%; 95% CI = 2.1, 9.3) and trend (increase per month after intervention) (1.0% per month; 95% CI = 0.6, 1.5) of on-time appointments (Table 2). We observed similar changes in the rates of patients attending the clinic within 3 days of their scheduled appointments, with an increase in level of attendance of 4.2% (95% CI = 1.6, 6.7), and a monthly trend increase of 0.8% (95% CI = 0.6, 1.1). Appointment-keeping at the control facilities did not change.

**Table 2:** Estimated changes in appointment keeping and medication gaps resulting from the intervention among experienced and newly treated patients

			**Pre-intervention trend (% per month)**		**Change in level (absolute)**		**Change in trend (% per month)**	
**Indicator**	Cohort	Group	Coefficient (± 95% CI)	p-value	Coefficient (± 95% CI)	p-value	Coefficient (± 95% CI)	p-value
**Keeping appointment or attending before appointment**	Exp.	Intervention	-0.25 (-0.54, 0.04)	0.096	**5.67 (2.07, 9.28)**	**0.002**	**1.04 (0.61, 1.47)**	**<0.001**
		Control	0.12 (-0.21, 0.45)	0.485	-2.78 (-7.72, 2.15)	0.268	0.64 (-0.05, 1.34)	0.071
		Difference	**-0.52 (-1, -0.04)**	**0.033**	**9.56 (-16.07, -3.04)**	**0.004**	0.48 (-1.29, 0.33)	0.248
	New	Intervention	-0.14 (-0.72, 0.43)	0.622	**8.75 (1.21, 16.29)**	**0.023**	-0.44 (-1.39, 0.51)	0.365
		Control	0.08 (-0.45, 0.61)	0.769	-1.8 (-9.84, 6.23)	0.660	0.85 (-0.16, 1.85)	0.100
		Difference	-0.72 (-1.7, 0.26)	0.147	**15.39 (-26.4, -4.38)**	**0.006**	-1.23 (-0.31, 2.76)	0.117
**Keeping appointment within 3 days**	Exp.	Intervention	-0.18 (-0.38, 0.01)	0.064	**4.16 (1.63, 6.69)**	**0.001**	**0.84 (0.58, 1.09)**	**<0.001**
		Control	0.31 (-0.15, 0.76)	0.186	0.49 (-5.41, 6.4)	0.870	-0.12 (-0.88, 0.65)	0.765
		Difference	**-0.71 (-1.17, -0.25)**	**0.003**	**6.46 (1.35, 11.58)**	**0.013**	**0.96 (0.16, 1.77)**	**0.019**
	New	Intervention	0.23 (-0.44, 0.89)	0.503	2.68 (-5.34, 10.69)	0.513	0.02 (-1.35, 1.4)	0.973
		Control	0.23 (-0.12, 0.58)	0.194	-1.13 (-9.68, 7.43)	0.796	0.11 (-0.86, 1.08)	0.823
		Difference	-0.27 (-0.87, 0.33)	0.374	7.85 (-17.16, 1.45)	0.098	-0.31 (-0.8, 1.42)	0.585
**Medication gap more than 14 days**	Exp.	Intervention	-0.15 (-0.62, 0.32)	0.523	-11.3 (-22.72, 0.07)	0.051	0.18 (-1.09, 1.46)	0.778
		Control	-0.48 (-0.94, -0.02)	0.041	-0.68 (-6.03, 4.66)	0.802	0.13 (-0.62, 0.87)	0.739
		Difference	0.33 (-0.37, 1.02)	0.355	-10.64 (-22.62, 1.33)	0.081	0.06 (-1.22, 1.34)	0.931
	New	Intervention	-0.37 (-1.42, 0.67)	0.484	-8.24 (-19.13, 2.65)	0.138	0.9 (-0.52, 2.32)	0.212
		Control	0.05 (-0.44, 0.54)	0.830	-3.87 (-12.43, 4.69)	0.376	0.14 (-1.15, 1.42)	0.833
		Difference	-0.43 (-1.75, 0.90)	0.529	-4.37 (-17.6, 8.86)	0.517	0.77 (-1.15, 2.68)	0.434

Exp*.* = Experienced patients, New = Newly treated patients; Figures in bold show significant differences between intervention and control group.

The relative difference between the intervention and control groups based on the monthly difference in visit rates increased significantly in both level (+6.5; 95% CI = 1.4, 11.6) and trend (1.0% per month; 95% CI = 0.2, 1.8) following the intervention for experienced patients attending the clinic within 3 days of their scheduled appointments (Table 2).

Among newly treated patients, there was an immediate significant increase in percentage of patients attending clinic on time (8.8% 95% CI = 1.2, 16.3) in the intervention cohort, but no significant change in trend or any other outcome variables in this patient group (Table 2).

### Medicine gaps of more than 14 days

**Figure 4:**
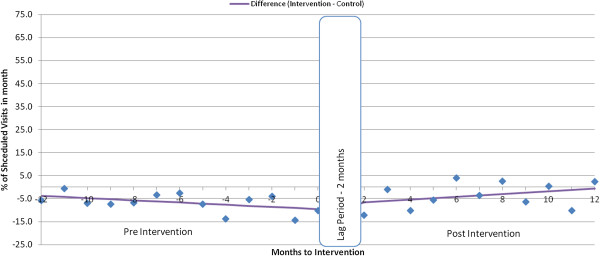
Difference in average % of visits kept within 3 days, experienced patients.

The level of experienced patients who had a gap in medication supply of more than 14 days (Figure 4) decreased following the intervention (-11.3%; 95% CI = -22.7, 0.1), but the decrease did not reach significance (p = 0.051), and the trend did not change. The newly treated cohort in the intervention sites and the control sites showed no significant changes in medication gaps after the intervention (Table 2).

### Self reported recalled perfect adherence

Over 90% of patients in the intervention and control groups recorded a self-reported perfect adherence during both the pre- and post-intervention periods, and no significant changes in either level or trend were found.

### Uptake of the intervention

Staff from all intervention facilities used the study tools successfully. The study team’s supportive supervision helped to resolve challenges, such as staff members not completely filling out the diary at the beginning of implementation. The diary was useful in determining who turned up for appointments and for initiating timely follow-up for clients who missed their appointments. Those who missed appointments were contacted and given more focused attention when they came back to the clinic, even if on their own volition. Providers used the revised MoH-257 patient record card and found the question on self-reported perfect adherence within the previous three days easy to administer to patients at the clinics. Clients who missed treatment doses were referred for more adherence counselling to help them overcome barriers to adherence and emphasize its importance.

Trained staff at the facilities calculated appointment-keeping indicators using a monthly summary sheet in the diary. During regular staff meetings to monitor intervention progress, the percentages for each of the appointment-keeping indicators were used to discuss facility performance. In addition, in some facilities that had not been holding such routine meetings, the intervention reinforced the practice.

## Discussion

Our findings show that it was possible to strengthen the appointment-keeping and performance-monitoring system in ART clinics in resource-constrained settings, and improve clinic attendance, through reviewing standardized appointment-keeping indicators during monthly facility management meetings. However, we were not able to show any improvement in adherence to treatment.

Among both experienced and newly treated patients in intervention facilities, rates of appointment-keeping consistently increased after the intervention compared to controls. The experienced patients had close to a significant decrease in medication gaps, which was sustained for 11 months after the start of the intervention. The level of changes (4.2–8.8%) may seem small, but should be seen in the context of the very large number of people on ART. If similar improvements could be achieved at the national level, between 18,000 and 38,000 patients would have a more favourable therapeutic outcome.

Standardized appointment-keeping indicators are useful in monitoring facility performance and promoting the use of routine data. The introduction of the appointment diary allowed service providers to periodically generate a list of patients who missed appointments. Failure to come to the facility for a scheduled visit provided an early warning that a patient was at risk of non-adherence [17]. Performance measurement raises awareness of existing practices and is an important driver for improvement [18]. The monthly facility summary enabled staff to track their performance level and motivated them to strengthen their practices. The limited use of available data to improve facility performance has previously been mentioned as a major challenge for many facilities in Kenya [14].

Patients need to attend appointments consistently to manage clinical issues related to ART and to avoid gaps in therapy [3], and coming to the clinic regularly has been significantly associated with optimum medication adherence [19]. Patients’ failure to attend the clinic when expected is an objective indicator that is easy to ascertain [9]; inconsistent attendance can identify patients requiring more focused interventions such as adherence counselling or outreach [16]. A common measure of ensuring patient retention in ART involves tracking patients who have missed their appointments [20,21]. Information from the appointment diary also enabled facilities to spread their workload by controlling the number of patients scheduled to visit on a given day.

The percentage of experienced patients with a gap in medication supply of more than 14 days decreased following the intervention, although the difference was not statistically significant. In this setting, client visits to the health facility are nearly always linked with an antiretroviral prescription refill, except when patients come early due to illness or unexpected travel. Antiretroviral medicines were generally available to patients in all facilities that participated in our study; thus, a decrease in medication gap would be linked to improved clinic attendance. An interruption of more than 14 days of therapy has been associated with a 50% probability of virological rebound among suppressed patients on a non-nucleoside reverse transcriptase inhibitor-based therapy, which predisposes them to developing resistance [22].

When implementing and scaling-up an innovation in a complex health system, knowledge about contextual factors is essential [23-25]. Therefore, in addition to the outcome measures presented here, we conducted interviews with representative members of the health staff and observed team meetings and interactions in the work places. Interview participants reported that the intervention allowed facilities to anticipate their daily workload and detect clients’ missing appointments, which helped them take timely action. In addition, information on individual patient attendance helped facility staff target counselling efforts and adjust the intervals of patient visits. Based on the interviews, the most important enabling factors appeared to be the involvement of key actors from each facility in planning, implementing, and monitoring; the positive attitude of health care workers; and a shift of staff members’ roles within the clinics. Task-shifting from health care providers to peer educators, social workers, and volunteers helped in the effort to track treatment defaulters, remind clients to keep appointments, counsel patients, and link patients to community health workers, while giving health care providers more time to implement the intervention [Unpublished data from qualitative part of the study]. Implementation constraints included poor coordination and weak management structures within the facilities, high staff turnover, lack of funds to follow-up patients who missed appointments, and staff shortages.

In considering national scale-up of this intervention, we would recommend: paying attention to local contexts, incentives, and institutions; anticipating unintended consequences; and developing and implementing programs that engage key actors by using data for ongoing problem-solving [23].

### Methodological considerations

The availability of routine data on clinic attendance and dispensed medicine enabled us to use interrupted time series analysis to demonstrate the impact of the intervention. The use of time series displays has been recognized as a robust method of identifying and visualizing patterns in complex systems [23]. Segmented regression analysis of interrupted time series data makes it possible to assess, in statistical terms, how much an intervention changed an outcome of interest immediately and over time, instantly or with delay, transiently or long-term, and whether factors other than the intervention could explain the change [26]. Changes can be associated and attributed to specific interventions at specific points. Our study’s use of control facilities and staggered intervention minimized the risk of external confounders.

The intervention and analysis were at the system level; we did not investigate individual patient characteristics and how they affect adherence. Medical records at facilities had limited demographic data, which could have been useful in exploring adherence at the patient level. In addition, data on opportunistic infections were not consistently available. Records on the quantities of medicines dispensed from the pharmacy were occasionally missing. In these instances, we imputed the quantities using an algorithm based on observed clinic norms at individual facilities. We could not perfectly match intervention and control facilities as demonstrated by differences in baseline characteristics, a common situation in group randomized trials with small numbers of groups.

Notably, the differences in baseline characteristics between study and control facilities that were observed regarding WHO Staging (mostly based on clinical staging) and CD4 values at start of treatment indicate that the intervention facilities had patients who appeared to be sicker at the initiation of therapy. Future studies should determine if sicker patients actually have poorer appointment keeping trends even when on maintenance phase (after 6 months of therapy).

Although the number of intervention facilities was small (n = 6), the analysis methods used are robust for the aggregated data. However, we are unable to statistically compare one facility to another due to the relatively small sample sizes at each facility. Because we sampled facilities with between 100 and 500 patients on ART, we are not sure if our results would apply to facilities with higher or lower number of registered patients.

At the end of the study 272 of 446 of patients were still in care at the intervention arm, and 304 of 352 patients in the control arm representing an attrition rate of 39% and 14% respectively. Statistical comparison of baseline characteristics of the subset identified as dropped out, versus those that remained till the end of the study in the intervention arm demonstrated that the subset that dropped out was not systematically different from the one that remained. Therefore, we believe that the potential bias arising from the differential dropouts in the intervention and control arms is of limited importance, although we cannot exclude the possibility that other factors may be responsible for the observed differences. In some of the patients’ clinical files, dropout was cited to be due to transfer out to other facilities, death, and loss to follow up. However, we were unable to compute the specific contribution of each reason cited, because the reason for loss to follow-up was not specified in most of the files.

## Conclusion

This study demonstrated that a facility-based intervention could strengthen the appointment-tracking and performance-monitoring system and that the use of facility-generated indicators was feasible and built the staff’s capacity to help patients improve their clinic attendance. For this approach to be scaled-up to the national level, the local context and the complex nature of the health system should be considered.

## Competing interests

The authors declare that they have no competing interests.

## Authors’ contributions

All study authors designed the study proposal and the tools for data collection. DK, SN, PB, PN, CA, and LG participated in data collection. All of the authors contributed to the data analysis and interpretation of the data summarized in the manuscript as well as in the writing the manuscript. All authors read and approved the final manuscript.

## Authors’ information

International Network for Rational Use of Drugs–Initiative on Antiretroviral Adherence.

## Pre-publication history

The pre-publication history for this paper can be accessed here:

http://www.biomedcentral.com/1472-6963/13/242/prepub

## Supplementary Material

Additional file 1 Modified MoH 257 for Adherence Intervention Study. Click here for file

## References

[B1] National AIDS Control Council United Nations General Assembly Special Session on HIV and AIDS 2010: Country Report—Kenya 2010 Nairobi, Kenya: National AIDS Control Council

[B2] UNAIDS Global HIV/AIDS Response—Epidemic Update and Health Sector Progress towards Universal Access—Progress Report 2011 2011 Geneva: World Health Organization

[B3] Chalker J Andualem T Gitau L Ntaganira J Obua C Tadeg H Waako P Ross-Degnan D Measuring adherence to antiretroviral treatment in resource-poor settings: the feasibility of collecting routine data for key indicators BMC Health Serv Res 2010 10 43 10.1186/1472-6963-10-43 20170479 PMC2836318

[B4] WHO Towards Universal Access, Scaling up Priority Interventions in the Health Sector; Progress Report 2008 Geneva: World Health Organization

[B5] Bangsberg DR Perry S Charlebois ED Clark RA Roberstson M Andrew R Moss A Non adherence to highly active antiretroviral therapy predicts progression to AIDS AIDS 2001 15 1181 1183 10.1097/00002030-200106150-00015 11416722

[B6] Wise J Operario D Use of electronic reminder devices to improve adherence to antiretroviral therapy: a systematic review AIDS Patient Care STDS 2008 22 6 495 504 10.1089/apc.2007.0180 18462071

[B7] Park WB Choe PG Kim SH Jo JH Bang JH Kim NJ Oh M Choe KW One year adherence to clinic visits after highly active antiretroviral therapy: a predictor of clinical progress in HIV patients J Intern Med 2007 261 268 275 10.1111/j.1365-2796.2006.01762.x 17305649

[B8] Gusdal AK Obua C Andualem T Wahlström R Tomson G Peterson S Ekström AM Thorson A Chalker J Fochsen G on behalf of the INRUD-IAA project Voices on adherence to ART in Ethiopia and Uganda: A matter of choice or simply not an option? AIDS Care 2009 21 11 1381 1387 10.1080/09540120902883119 20024714

[B9] Ross-Degnan D Pierre-Jacques M Zhang F Tadeg H Gitau L Ntaganira J Balikuddembe R Chalker J Wagner A Measuring adherence to antiretroviral treatment in resource-poor settings: the clinical validity of key indicators BMC Health Serv Res 2010 10 42 10.1186/1472-6963-10-42 20170478 PMC2834585

[B10] Bisson GP Gross R Bellamy S Chittams J Hislop M Regensberg L Frank I Maartens G Nachega JB Bisson GP Gross R Bellamy S Chittams J Hislop M Regensberg L Frank I Maartens G Nachega JB Pharmacy refill adherence compared with CD4 count changes for monitoring HIV-infected adults on antiretroviral therapy PLoS Med 2008 5 5 e109 10.1371/journal.pmed.0050109 18494555 PMC2386831

[B11] Patterson D Swindells S Mohr J Adherence to protease inhibitor therapy and outcomes in patients with HIV infection Ann Intern Med 2001 134 62511281750

[B12] Mills EJ Nachega JB Buchan I Orbinski J Attaran A Singh S Rachlis B Wu P Cooper C Thabane L Wilson K Guyatt GH Bangsberg DR Adherence to antiretroviral therapy in sub-Saharan Africa and North America: a meta-analysis JAMA 2006 296 6 679 690 10.1001/jama.296.6.679 16896111

[B13] Bangsberg D Less than 95% adherence to nonnucleoside reverse transcriptase inhibitor therapy can lead to viral suppression Clin Infect Dis 2006 43 939 941 10.1086/507526 16941380

[B14] National AIDS/STI Control Programme Annual Health Sector HIV Report 2009: Progress with the National Health Sector Response 2010 Nairobi: National AIDS and STI Control Program

[B15] Njogo SM Improving Appointment Keeping and Adherence Monitoring In ART Facilities in Kenya: Views of Providers and Patients Presentation at the Third International Conference for Improving Use of Medicine, Antalya, Turkey, November 2011 http://www.inrud.org/ICIUM/ConferenceMaterials/544-njogo-_c.pdf (accessed 29 May 2012)

[B16] Chalker J Wagner A Tomson G Laing R Johnson K Wahlstrom R Ross-Degnan D on behalf of INRUD-IAA Urgent need for coordination in adopting standardized antiretroviral adherence performance indicators JAIDS 2010 53 2 159 161 10.1097/QAI.0b013e3181befa12 19861899

[B17] Chalker J Andualem T Minzi O Ntaganira J Ojoo A Waako P Ross-Degnan D Monitoring adherence and defaulting for adherence therapy in five east African countries: an urgent need for standards JIAPAC 2008 7 193 199 18626124 10.1177/1545109708320687

[B18] Rhydderch M Elwyn G Marshall M Grol R Organisational change theory and the use of indicators in general practice Qual Saf Health Care 2004 13 213 217 10.1136/qshc.2003.006536 15175493 PMC1743845

[B19] Kunutsor S Walley J Katabira E Muchuro S Balidawa H Namagala E Ikoona E Clinic attendance for medication refills and medication adherence amongst an antiretroviral treatment cohort in Uganda: a prospective study AIDS Res Treat 2010 2010 872396 21490907 10.1155/2010/872396 PMC3065731

[B20] Assefa Y Kiflie A Tesfaye D Haile Mariam D Kloos H Edwin W Laga M Van Damme W Outcomes of antiretroviral treatment program in Ethiopia: retention of patients in care is a major challenge and varies across health facilities BMC Health Serv Res 2011 11 81 10.1186/1472-6963-11-81 21501509 PMC3094207

[B21] Messou E Weinstein MC Dabis F Freedberg KA Cost-effectiveness of preventing loss to follow-up in HIV treatment programs: a Cote d’Ivoire appraisal PLoS Med 2009 6 10 e1000173 10.1371/journal.pmed.1000173 19859538 PMC2762030

[B22] Gardner EM Burman WJ Steiner JF Anderson PL Bangsberg DR Antiretroviral medication adherence and the development of class-specific antiretroviral resistance AIDS 2009 23 1035 1046 10.1097/QAD.0b013e32832ba8ec 19381075 PMC2704206

[B23] Van Damme W Kober K Kegels G Scaling-up antiretroviral treatment in Southern African countries with human resource shortage: how will health systems adapt? Soc Sci Med 2008 66 2108 2121 10.1016/j.socscimed.2008.01.043 18329774

[B24] Paina L Peters D Understanding pathways for scaling up health services through the lens of complex adaptive systems Health Policy Plan 2011 1 9 http://healpol.oxfordjournals.org/content/early/2011/08/05/heapol.czr054.full.pdf+html 10.1093/heapol/czr054 21821667

[B25] Savigny D De, Adam T Systems Thinking for Health Systems Strengthening 2009 Geneva: WHO

[B26] Wagner AK Soumerai SB Zhang F Ross-Degnan D Segmented regression analysis of interrupted time series studies in medication use research J Clin Pharm Ther 2002 27 299 309 10.1046/j.1365-2710.2002.00430.x 12174032

