# Supplementary material for: Facility-level intervention to improve attendance and adherence among patients on anti-retroviral treatment in Kenya—a quasi-experimental study using time series analysis
Source: BMC Health Serv Res. 2013 Jul 1;13:242. doi: 10.1186/1472-6963-13-242 (PMC3704969; doi:10.1186/1472-6963-13-242)
Supplement: Additional file 1 — Modified MoH 257 for Adherence Intervention Study. [file 1472-6963-13-242-S1.doc]

**Appendix 1: Modified MoH 257 for Adherence Intervention Study**
